# Editorial: Cigarette Smoke, E-Cigarette/E-Vaping and COVID-19: Risks and Implications in This New Era

**DOI:** 10.3389/fphys.2021.724910

**Published:** 2021-09-09

**Authors:** Blanca Camoretti-Mercado, Qianjin Liao, Zhi Tian, Diane Allen-Gipson

**Affiliations:** ^1^Department of Internal Medicine, Morsani College of Medicine, University of South Florida, Tampa, FL, United States; ^2^Huan Cancer Hospital and the Affiliated Cancer Hospital of Xiangya School of Medicine, Central South University, Changsha, China; ^3^Department of Pharmaceutical Sciences, USF Health Taneja College of Pharmacy, University of South Florida, Tampa, FL, United States

**Keywords:** tobacco smoking, COVID-19, alcohol, inflammation, SARS-CoV-2, vaping, electronic cigarette, ACE2

In December 2019, a cluster of pneumonia was reported in Wuhan, China. The new disease, COVID-19, is caused by the novel coronavirus SARS-CoV-2 and rapidly spread to become pandemic in March 2020. Worldwide, on August 4, 2021, confirmed cases and deaths exceed 199 and 4 million, respectively, and there are 548,167 new cases and over 3 billion vaccine doses administrated (https://covid19.who.int). Cigarette smoking (CS) is a universal public health concern too. Because exposure to CS increases the incidence and mortality from infections (Jiang et al., 2020), CS could rise the risk for COVOD-19. Yet, the relationship between smoking and COVID-19 has been controversial. Lower prevalence in hospitalized COVID-19 patients (Goyal et al., 2020) and lower risk of SARS-CoV-2 infection (Lee et al., 2021) were reported among smokers. However, a meta-analysis (WHO, 2020) suggests an association between smoking and COVID-19 severity outcomes. The U.S. Centers for Disease Control and Prevention and the Food and Drug Administration list smoking as a factor likely to make COVID-19 worse. This Research Topic presents new findings and reviews evidence on the role of smoking/vaping on COVID-19 prognosis, diagnosis, and outcomes.

Neither smoking tobacco nor vaping have health benefits. In fact, both damage the respiratory, cardiovascular, and other organ systems. Furthermore, smoking often occurs with drinking. About 15% of ingested alcohol is exhaled or metabolized in the lung where it impairs neutrophil recruitment and compromises epithelial barrier function (Sisson, 2007; Muthumalage et al., 2019). In a study of 1,500+ COVID-19 patients with smoking or alcohol consumption histories, Dai et al. show more severe COVID-19 and higher probability of death in smokers than non-smokers. While alcohol consumption is not associated with severity or mortality in this study, Wetzel and Wyatt find that e-cigarette users are at high risk for binge and chronic alcohol consumption. They postulate that dual vs. single substance use might promote tissue damage, decrease immune response and pathogen clearance, and increase IL-6 and IL-8 production and inflammation, which would elevate COVID-19 complications risk. Xie et al. highlight the importance of smokers' and alcohol consumers' behaviors that facilitate the spread of viruses. Peaks in COVID-19 cases and deaths in U.S. occurred after the (premature) opening in summer 2020 and the holidays in January 2021 (https://covidtracking.com/data/charts/2-metrics-7-day-average-curves). These events likely promoted physical closeness and crowding while drinking, hand-to-mouth contact, and smoke-induced lung insult that might increase susceptibility to COVID-19. This hypothesis could be tested by comparing with data during periods of lockdowns and social distancing. Xie et al. note that isolation and its negative effect on mental health make quitting smoking difficult and contribute to smoking and drinking.

Some COVID-19 patients suffer long-COVID-19, which manifests as symptoms that last weeks or months after being infected with SARS-CoV-2 or can appear weeks after infection. The cause and role of smoking in long COVID-19 is unknown. Compared to non-smokers, Kaur et al. find persistent high levels and activity of ACE2, the SARS-CoV-2 receptor, and of eotaxin and monocyte chemoattractant protein-1 in the sera of COVID-19 patients with smoking history. The relevance of these alterations on long-COVID-19 deserves attention.

Vaping, the inhalation of e-cigarette aerosols, associates with lung inflammation and injury. Teenagers and young adults who vape and/or smoke vs. non-users are 2.6–9 times more likely to obtain a COVID-19 test because of experiencing symptoms (Gaiha et al., 2020). Furthermore, youth who ever used e-cigarettes alone or plus conventional cigarettes are 5 or 6.8 times more likely, respectively, to receive a COVID-19 diagnosis than their peers who did not use. In this Research Topic, Masso-Silva et al. show changes in lung gene expression of mice exposed to e-cigarette aerosols that indicate neutrophil activation, a potential driver of acute lung injury in COVID-19. They also show vaping mint increases expression of ACE2. Moreover, Sivaraman et al. demonstrate that pre-exposure to vaped e-liquid vs. vehicle alone results in worse pulmonary inflammation and mortality in mice infected with the murine-tropic coronavirus MHV-A59. Together, these preclinical investigations indicate that vapers could have augmented susceptibility to SARS-CoV-2 infection and be at higher risk of developing severe COVID-19.

The presence of comorbidities and the relatively small studies' sample size had limited the generation of robust conclusions about COVID-19 and smoking association. Our Research Topic contains four reports with variable number of patients. In a study of 622 COVID-19 patients at an academic hospital in China, Peng et al. find that smokers have higher mortality risk than non-smokers after adjusting for age, sex, and underlying diseases. Smokers exhibit greater levels of leucocytes, hemoglobin and creatinine, and lower erythrocyte sedimentation rate than non-smokers. Zhong et al. study 91 patients between 3 months and 76 years of age in a tertiary hospital in China during the first 3 months of the outbreak. Severe disease occurs in people 65 or older, married, and with or below primary school education. While smoking, drinking, or betel quid chewing have no significant relationship with disease severity, diabetes and being retired/unemployed associate with worse COVID-19. In a longitudinal study of 10 controls and 25 COVID-19 adults with no comorbidities in Brazil, Alberca et al. show no difference in the number of leucocytes among current or former smokers and COPD patients during the hospital stay. However, smokers and COPD patients are at risk of kidney injury, and death occurs only among smokers. Although small, this study shows that, in the absence of comorbidities, smoking or COPD influences COVID-19 course. Replicating these results in larger populations and different demographics would be compelling. Together, these studies reveal that COVID-19 might present differently in distinct settings or countries or phases of the pandemic.

Children carry and transmit SARS-CoV-2. Compared to adults, COVID-19 in children is milder with lower hospitalization rates and death in U.S. and Europe. Potential protective factors are the transfer of maternal antibodies and antiviral proteins in the milk (Root-Bernstein, 2020a), administration of certain vaccines (Root-Bernstein, 2020b), lower ACE2 expression (Molloy and Bearer, 2020), and absence of underlying diseases in general. However, children are exposed to secondhand or direct CS, which may deteriorate pre-existing conditions or lead to respiratory problems and infections in at-risk patients. Schiliro et al. review that pediatric COVID-19 cases continue to increase, its symptoms are non-specific, and about 50% of cases happen with respiratory coinfections. While 80% of North America's pediatric intensive care unit admissions have developmental delay and/or genetic anomalies, only 4% suffer from chronic lung disease such as asthma. Nevertheless, multisystem inflammatory syndrome in children resembling Kawasaki disease can develop after COVID-19 diagnosis.

The course of the pandemic will shift toward improved outcomes as more people worldwide are vaccinated, effective therapies developed, and the mechanisms of disease understood. Unfortunately, new virus variants emerge, and old and new inhalation modalities are adopted with unknown health consequences. Thirupathi et al. show that physical exercise attenuates lung and muscle inflammation and histopathology in mice chronically exposed to hand-rolled cornhusk cigarette smoke. Elegant pre-clinical studies by Tian et al. elucidate the role of the CD73/adenosine A_2B_ receptor/PKCa/ERK pathway in cigarette smoke-mediated airway epithelial injury.

In conclusion, this Research Topic provides a view on how cigarette smoke, e-cigarette, or vaping can predispose and worsen COVID-19 and the negative effect of dual use of smoking with alcohol on this disease. Gaps in knowledge will close as preclinical and clinical investigations are completed and new studies implemented. ClinicalTrials.gov lists seven interventional or behavioral studies on COVID-19 and either smoking or tobacco use or nicotine. No study on COVID-19 and e-cigarette/vaping is registered. A study on immune senescence association with prior smoking as a susceptibility factor in COVID-19 is underway (NCT04403386), Longitudinal studies are necessary to understand long-COVID and develop appropriate treatments. Success of COVID-19 vaccination globally will reduce the risk of SARS-CoV-2 infection and spread of COVID-19 and improve long-COVID-19 symptoms. Because achievement of these goals will take months if not years, every effort to quit smoking and vaping should be a priority.

## Author Contributions

All authors contributed to the manuscript and read and approved it for publication.

## Conflict of Interest

The authors declare that the research was conducted in the absence of any commercial or financial relationships that could be construed as a potential conflict of interest.

## Publisher's Note

All claims expressed in this article are solely those of the authors and do not necessarily represent those of their affiliated organizations, or those of the publisher, the editors and the reviewers. Any product that may be evaluated in this article, or claim that may be made by its manufacturer, is not guaranteed or endorsed by the publisher.
